# Phytochemical evaluation, nutritional and pharmacological values of *Trigonella foenum-graecum* plant: a comprehensive review

**DOI:** 10.3389/fchem.2026.1849521

**Published:** 2026-07-08

**Authors:** Bayansulu Otegenova, Gulzat Berganayeva, Bates Kudaibergenova, Aleksandra Szydłowska-Czerniak, Adewale Olufunsho Adeloye, Moldyr Dyusebaeva, Abdul Bari Shah

**Affiliations:** 1 Faculty of Chemistry and Chemical Technology, Al-Farabi Kazakh National University, Almaty, Kazakhstan; 2 Department of Analytical Chemistry and Applied Spectroscopy, Faculty of Chemistry, Nicolaus Copernicus University in Toruń, Toruń, Poland; 3 Department of Chemistry, College of Science, Engineering and Technology (CSET), University of South Africa, Florida, South Africa; 4 Research Center for Medicinal Plants, Al-Farabi Kazakh National University, Almaty, Kazakhstan

**Keywords:** antidiabetic potential, anti-inflammatory activity, antioxidant activity, cultivation, fenugreek, phytochemicals, sustainable agriculture, *Trigonella foenum-graecum*

## Abstract

The growing scientific interest in medicinal and nutritionally valuable plants has highlighted the importance of *Trigonella foenum-graecum* (fenugreek), a species widely recognized for its diverse phytochemical, nutritional, and pharmacological significance. This member of the legume family is an annual self-pollinating herb widely cultivated across Mediterranean, Asian, African, and other regions due to its adaptability to different climatic conditions and its ability to fix atmospheric nitrogen. It is also traditionally used in culinary and medicinal traditional practices, particularly as spice and functional food ingredients. This review provides an updated overview of the botanical characteristics, geographical distribution, cultivation conditions, phytochemical composition, nutritional value, and biological activities of the plant, with particular emphasis on recent advances in nanoformulations, biomedical applications, hemocompatibility, and recent pharmacological advances. The seeds and leaves contain proteins, dietary fibers, minerals, vitamins, and various bioactive compounds such as saponins, flavonoids, alkaloids, polyphenols, and galactomannans, which have been reported to exhibit antioxidant, antidiabetic, anti-inflammatory, anticancer, antilipidemic, antimicrobial effects, as well as low hemolytic activity. By integrating recent findings on fenugreek characteristics, phytochemistry, and biological activities, this review provides an updated perspective on its scientific relevance and ethnobotanical applications.

## 1 Introduction

The traditional use of medicinal plants, which dates back centuries, is characterized by a low incidence of side effects. In recent decades, the world has paid increasing attention to plant components, both as food and medicine. This growing trend is driven not only by the shortcomings of synthetic drugs, but also by the significant health potential of nature. Plants are valuable sources of structurally diverse secondary metabolites, such as alkaloids, flavonoids, saponins, terpenoids, and phenolic compounds. These substances not only nourish us, but also have many beneficial pharmacological effects (Bari Shah et al., 2026; Marrelli, 2021; Rizvi et al., 2022). Owing to these bioactive properties, herbal preparations can play an important role in the prevention and treatment of various chronic diseases, including hypertension, diabetes, atherosclerosis, cancer, and cardiovascular disease (Ansari et al., 2025). While synthetic drugs are often costly, and may not always produce the desired results, coupled with accompanied side effects; in contrast natural compounds are increasingly considered safer, more versatile, and cost-effective (Dar et al., 2023). In this context, particular attention is paid to plants that possess both high nutritional values and medicinal properties. One example is *Trigonella foenum-graecum*, a legume belonging to the Fabaceae (Leguminosae) family.

Fenugreek, also known as *T. foenum-graecum*, is a widely cultivated medicinal and culinary plant that is traditionally grown in various regions worldwide, including the Mediterranean, South Asia, the Middle East, and North Africa (Shahrajabian et al., 2021). The plant is classified in the genus *Trigonella*, meaning “little triangle” in Latin, referring to its yellowish-white flowers triangular in shape. Due to its adaptability to a wide range of climatic and soil conditions, fenugreek is cultivated globally and has attracted increasing agricultural and scientific interest. As a nitrogen-fixing legume, it enhances soil fertility and is increasingly recognized as a crop that support sustainable agriculture. In the culinary applications, fenugreek is valued as a spice that enhances flavor, color, and texture of food products. Its seeds have beneficial properties and are considered carminative, tonic, while the leaves serve as nutritious leafy vegetable (Sarwar et al., 2020). In traditional medicine, fenugreek is used as a remedy to facilitate childbirth and stimulate lactation in nursing mothers. In Egypt, it is traditionally employed to relieve menstrual pain. Fenugreek tea “hilba” is popular among tourists as a remedy for improving digestion (Singh et al., 2022). In Chinese medicine, fenugreek is traditionally used to treat cold-related diseases such as abdominal pain and hernia, due to its ability to “warm the kidneys” and fight colds (Zarghi et al., 2021). These properties indicate the significant nutritional value and functional role of fenugreek in both dietary nutrition and folk medicine.

Recent research indicates that fenugreek exhibits diverse biological activities, particularly with relation to metabolic regulation, inflammatory processes, microbial infections, and cancer-related mechanisms, as demonstrated by both laboratory studies and human clinical trials (Ahmad et al., 2025). Fenugreek seeds contain a variety of bioactive compounds that contribute to their pharmacological activities. In particular, their antioxidant activity is largely attributed to phytochemical such as phenolic compounds which are also considered important in modulating physiological processes and reducing oxidative stress (Fouda et al., 2024). Among the bioactive constituents of fenugreek seeds, the steroidal saponin diosgenin has attracted considerable attention due to its reported role in cancer chemoprevention and metabolic regulation (Sen et al., 2025). Phytochemical studies using techniques as gas chromatography-mass spectroscopy (GC-MS) and spectroscopic methods have revealed the presence of numerous terpenoids and flavonoids, which are linked to potential anticancer activity (Qadir et al., 2022). Furthermore, specific groups of phytochemicals also have been associated with other biological effects. Saponins, flavonoids, alkaloids and amino acid derivatives (2S, 3R, 4S)-4-hydroxyisoleucine (4-HIL) have been associated with antidiabetic and antilipidemic properties due to their effect on glucose homeostasis and lipid metabolism (Haxhiraj et al., 2024). Phenolic compounds and steroidal saponins are considered key contributors to anti-inflammatory and antibacterial activities (Jayaraman and Ramasamy, 2024), while galactomannan-rich polysaccharides have been associated with low hemolytic activity and potential biomedical applications (Tahmasebi and Mohammadi, 2025).

This review present a comprehensive overview of the nutritional, phytochemical, and pharmacological properties of *T. foenum-graecum*. Particular emphasis is placed on biologically active substances, traditional methods of application and therapeutic effects as demonstrated in experimental and clinical studies. Thus, fenugreek represents a promising medicinal and functional plant that requires further study for broader application in modern medicine and nutrition sciences.

## 2 Methodology

All relevant information regarding the botanical characteristics, nutritional, phytochemical, and pharmacological properties of *T. foenum-graecum* was collected from published scientific literature. Research articles were retrieved from major scientific databases, including PubMed, Web of Science, Scopus, Google Scholar, and ScienceDirect. Publications were selected based on relevance by evaluating titles, abstracts, and keywords. Chemical structures of bioactive compounds were drawn using ChemDraw 22.2.0 and BioRender, along with Microsoft PowerPoint.

## 3 Botanical characteristics, origin and cultivation of *Trigonella foenum-graecum*

### 3.1 Botanical perspective of fenugreek

**FIGURE 1:**
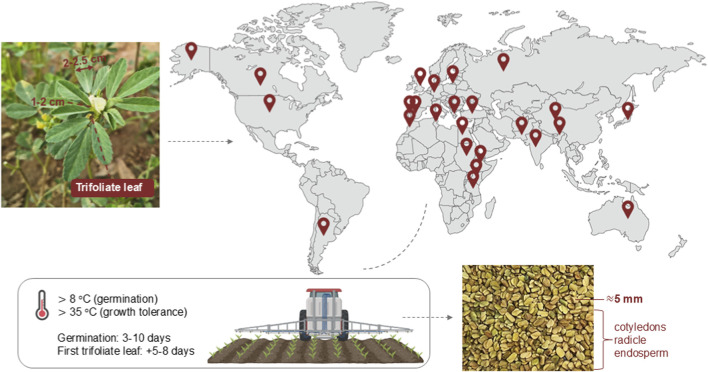
Botanical features, geographical distribution and cultivation of *Trigonella foenum-graecum*.

Fenugreek (*Trigonella foenum-graecum*) is an annual dicotyledonous belonging to the subfamily Papilionaceae of the legume family (Fabaceae). It grows optimally under mild Mediterranean climatic conditions. The plant reaches maturation approximately in 4 months. The herb fenugreek typically flowers in midsummer. Fenugreek, as illustrated in Figure 1, features a branched stem, trifoliate leaves, yellowish-white flowers, root nodules, and seeds of a golden-yellow color (Acharya et al., 2006). A branched, upright herb, which typically grows to 30–60 cm and varying by type (Yadav and Baquer, 2014). Fenugreek has pinnately compound leaves comprising three leaflets, each measuring approximately 2–2.5 cm and nodulated roots. The plant develops white to yellow flowers, 1–2 cm in size, 30–40 days after sowing, followed by thin, curved pods (3–15 cm) holding golden-yellow seeds (Ruwali et al., 2022 **)**. The plant also has a strong, spicy aroma that is which readily adheres to the hands and persists after washing (Singh et al., 2025a). As a self-pollinating annual legume, fenugreek matures in about 4 months. Its productivity can be greatly enhanced by good farming techniques, including irrigation, pest control, and timely gathering (Pardhe and Jawale, 2022). Genotypic variation also plays an important role in determining the agronomic and biochemical properties of fenugreek. Recent comparative analysis of green-seeded and yellow-seeded genotypes demonstrated significant differences in plant height, branching, flowering time, maturity, seed yield, and phytochemical composition (Singh et al., 2025b).

The seeds are small (around 5 mm), hard, angular, and yellowish-brown with an oval-rhomboid shape; they are suppurative, aperient, and diuretic (Pal and Mukherjee, 2020). A small indentation on one long side contains the hilum and micropyle, the hilum appearing as a whitish dot (Zadeh et al., 2015). This indentation forms a diagonal furrow, splitting the seed into two unequal lobes. In cross-section, two yellowish cotyledons are located in the larger lobe, while the radicle is present in the smaller lobe; both are surrounded by a darker, translucent white endosperm (Pardhe and Jawale, 2022).

Fenugreek is predominantly a self-pollinating plant, as its flowers often stay closed while the petals fit tightly together (Mehrafarin et al., 2011). Even for those types of fenugreek where the flowers are opened, self-pollination is still the main way they reproduce. Scientific data indicates that, due to the unique structure and physiological characteristics of the flower, fenugreek is capable of self-fertilization even when its flowers are open (Acharya et al., 2006). Legumes in which more than 10% of flowers are pollinated by pollen from other plants are considered cross-pollinated (Zadeh et al., 2015). Fenugreek belongs to species with rare cross-pollination (Ruwali et al., 2022). This is attributed to protogyny, where the stigma matures earlier than the anthers. For successful breeding and obtaining fenugreek hybrids, breeders use the method of artificial pollination (Mehrafarin et al., 2011). They pollinate closed flowers at an early stage of development, when the stigma is already receptive pollen, but the anthers have not yet opened and are located below the stigma (Zadeh et al., 2015). Flowering shoots of fenugreek can be divided into two types: normal shoots, characterized by indeterminate growth and produce flowers in the leaf axils (Mehrafarin et al., 2011). In contrast, the blind shoots bear both axillary and terminal flowers, acting as apex carriers for the formation of seed pods. Among fenugreek flowers, there are both closed (cleistogamous) and open (aneictogamous) forms, however, the closed flowers predominate (Deng et al., 2023).

### 3.2 Origin and geographical distribution

Fenugreek is a plant well adapted to diverse climatic conditions and widespread in the regions adjacent to the Eastern Mediterranean (Choudhary et al., 2024). *Trigonella* species are found on all continents except Antarctica (Acharya et al., 2006). Initially, fenugreek was used as a forage crop in the Mediterranean, North Africa, and the Middle East. Over time, its cultivation expanded to cover the Indian subcontinent and most of South Asia (Giridhar et al., 2023). Fenugreek is cultivated extensively in several countries worldwide. Major production occurs in South Asian countries such as India, Pakistan, Iran, Afghanistan, and Nepal (Figure 1). It is widely grown in North Africa and the Middle East, including Egypt, Morocco, Algeria, Turkey, and Lebanon. In Europe, cultivation is reported in countries such as France and Spain. Additionally, fenugreek is produced in China, Canada, Argentina, the Unites States, and Australia (Ruwali et al., 2022). Commercial cultivation is practiced in India, Ethiopia, Morocco, Egypt and other countries where high-yielding varieties are used (Ahmad et al., 2023). Australia’s active interest in fenugreek production has led to its gradual establishment as a vital producing nation, with new cultivars under development. The largest importers of fenugreek seeds are Japan, Saudi Arabia, Korea, Sri Lanka, and the United Kingdom (Giridhar et al., 2023).

Fenugreek is referenced in the ancient Egyptian Ebers papyrus (1500 BCE) as a medicinal plant used for therapeutic purposes since ancient times (Ruwali et al., 2022). The natural habitat of wild fenugreek spans a wide area, including Punjab and Kashmir regions of India and Pakistan, Mesopotamia and Persia, Asia Minor, and parts of Southern Europe (Mehrafarin et al., 2011). Despite this broad geographical presence, the evidence suggests that Asia is the most probable location where fenugreek first appeared (Acharya et al., 2006). Its limited presence in Southern Europe and disappearance from regions such as Sicily, Ischia and the Balearic Islands supports the idea that its roots are elsewhere (Mehrafarin et al., 2011). Fenugreek may serve as an important nutritional source in countries with constrained healthcare resources, specifically for individuals with limited access to costly medications (Neelakantan et al., 2014).

Fenugreek is currently being explored in various regions globally. The plant is recognized by numerous names in various languages. For instance, it's called *Fenugrec* in French, *Methi* in Hindi, *Bockshorklee* in German, *Fienogreco* in Italian, *Pazhitnik* in Russian, *Alholva* in Spanish, *Koroha* in Japanese, *Hulba* in Arabic, *Halba* in Malay, and *K’u-Tou* in Chinese (Pal and Mukherjee, 2020). New agricultural initiatives are introducing it in Asia, Africa, the Americas and European Union. These areas possess climates and conditions favorable for their growth. The goal is to identify fenugreek as a potential alternative crop, helping to address issues in agricultural production caused by global warming and climate change (Giridhar et al., 2023).

### 3.3 Cultivation

Fenugreek is well adapted to diverse climatic, temperatures, and soils, and is now cultivated in more than 20 countries worldwide (Chaudhary et al., 2018). With improvements in the breeding practices and cultivation techniques, fenugreek productivity per hectare has significantly increased. In the past the yield of 1 ton of seeds per hectare was an indication of a high result, whereas, today it often reaches over 2 tons (Mehrafarin et al., 2011). Thus, the yield of fenugreek seeds ranges from 500–3320 kg/ha, demonstrating considerable economic profitability. Other countries also show various improvements in cultivation (Mehrafarin et al., 2011). In general, high yields of fenugreek directly depend on the correct approach to its cultivation and the use of effective agronomic techniques.

The timing of planting of fenugreek in rural areas is determined by the precipitation regime. It is advisable to plant the crop at the end of the rainy season, since it mainly uses residual soil moisture as the crop is sensitive to excessive moisture during the growing season (Giridhar et al., 2023). Crop yield directly depends on soil moisture availability throughout the growing season. Studies have shown that a lack of moisture leads to a decrease in the yield of diosgenin. At the same time, the application of an irrigation regime with a depletion of available soil moisture by 35% contributes to an increase in diosgenin yields compared with traditional irrigation, which indicates a positive reaction of the crop to moderate watering (Mehrafarin et al., 2011).

Fenugreek is particularly demanding of the temperature regime, its germination requires temperatures above 8 °C, and during the growing season it can withstand air temperatures above 35 °C. Humidity requirements are moderate (slightly higher and remains constant during sunrise), well-drained soils with a pH between 5.3 and 8.2 (Vanghele et al., 2020). Heavy and moist soils limit the growth of this crop in arid areas and the interest in growing fenugreek in temperate climates has increased due to its adaptation to arid conditions (Chowdhury et al., 2014; Mehrafarin et al., 2011).

Fertilizers are crucial for the successful cultivation of fenugreek, as they support important plant processes such as metabolism, energy transfer at the cellular level, photosynthesis, and respiration (Khan et al., 2014). Fenugreek has the greatest need for N and K, the least for Ca, and a moderate for P (Acharya et al., 2006). The addition of magnesium enhanced the effect of the doubled nitrogen allowance, but the highest yields were obtained with the addition of Ca (Mehrafarin et al., 2011). Applying Ca alone had a greater positive impact on seed harvest than applying Mg alone. The maximum viscosity was observed with the addition of Ca and Mg with twice the N content (Vanghele et al., 2020). Studies have shown that fertilizers containing nitrogen, phosphorus, and potassium positively affect fenugreek seed yields, and improve hay quality (Salehi et al., 2017). Experiments in pots have shown that the maximum seed yield is achieved with double application of N, P and K in combination with Ca and Mg (Tura et al., 2023). It is important that the soil is rich in nutrients and Ca, which reduces the application of nitrogen fertilizers (Getu et al., 2022). Without the addition of Ca and Mg, the concentration of diosgenin increased as much as possible with a doubling of the nitrogen norm (Pardhe and Jawale, 2022). An inverse relationship was found between nitrogen uptake and diosgenin content (Mehrafarin et al., 2011). To avoid salt accumulation in the soil, it is recommended to apply fertilizers in small doses, rather than large ones at long intervals (Salehi et al., 2017). It is also recommended to avoid growing other legumes on the same soil as fenugreek for 2–3 years (Vanghele et al., 2020).

The use of inorganic nitrogen fertilizers is associated with environmental risks associated with leaching and surface runoff, which can lead to eutrophication of reservoirs and the growth of pathogenic microflora (Acharya et al., 2006). The inclusion of fenugreek in agricultural systems contributes to the sustainable development of the agricultural sector, reducing producer costs for fertilizers and responding to public demand for environmentally oriented methods. A decrease in the amount of fertilizers applied correlates with a decrease in the level of surface water pollution (Verma et al., 2016).

The germination of fenugreek seeds in a soil environment usually takes from 3 to 10 days. The formation of the first simple leaf in seedlings is observed 6–10 days after the emergence of seedlings. Due to the nitrogen-fixing nature of fenugreek as a legume plant, inoculation with a suitable *Rhizobium spp* strain is required to optimize its growth potential (Mehrafarin et al., 2011). It should be noted that if there is a natural population of these bacteria in the soil, the seed inoculation procedure becomes redundant. After inoculation, bacteria invade the fenugreek root system, followed by the formation of root nodules, in which an active process of fixation of atmospheric nitrogen is carried out.

## 4 Nutritional application of *Trigonella foenum-graecum*

Fenugreek is globally consumed both as a culinary ingredient and as a medicinal plant. In human nutrition, the seeds are widely used as food products, culinary spices, ingredient and as functional components of traditional diets (Sen et al., 2025). Fenugreek containing foods, particularly when combined with vitamin-C rich ingredients, may potentially be employed to improving iron intake (Khoja et al., 2021). In India, seed powder is commonly used for indigestion, while cold water extracts known as “fenugreek tea” are taken for breathing disorders such as lung infections. Furthermore, the seed powder and extracts are long used for treating skin problems such as boils, abscesses, thermal injuries, and other important uses as hair tonics, conditioners, and lactation supporting agents, hormonal imbalance, and diarrhea. Its seeds are used in African cuisine to enrich bread, as their galactomannan provides soluble dietary fiber that improves nutritional value and bread stability. The leaves and seeds are also widely used to flavor foods such as curries, pickles, cheeses, seasonings, and chutneys, while green leaves can be eaten fresh and the seeds are ground into powders or pastes for cooking. Kachi, a traditional Iranian dish for nursing mothers, is prepared using the seeds of fenugreek together with butter, sugar, and flour, along with various spices (Akhtari et al., 2024). In traditional Iranian and Indian practices, fenugreek is consumed as powders, infusions, or decoctions (Singh et al., 2022). It is also used in imitation maple syrup because of its maple-like aroma and flavor. In the Middle East and Southeast Europe, fenugreek has traditionally been used to relieve menstrual and labor abdominal pain. In Egypt and Ethiopia, it is commonly added to bread, while in Switzerland it is used to flavor cheese, and in the United States it is mainly included in spice mixtures for soups and stews. Also reported to support lymphatic cleansing activity by helping deliver nutrients to cells and remove waste products from the body (Wani and Kumar, 2018).

The seed gum is commonly used to replace fat in various food products (Kaur et al., 2025). For example, adding 0.05%–0.5% fenugreek seed gum to low-fat beef burgers improved fat retention and reduced cooking shrinkage (Tabarestani et al., 2024).

Fenugreek gum from seeds is commonly applied in food formulations as a dietary fiber that enhances texture and regulate glycemic response (Wang et al., 2026). Steroidal-rich extracts are presented as promising candidate aimed at improving obesity and metabolic disorders (Sen et al., 2025). Fenugreek seed powder contains allergenic proteins, with reactions observed at doses as low as about 2 mg, and shows high cross-reactivity in patients with peanut allergy (Faeste et al., 2009). In a survey of spices sold in Saudi Arabian markets, fenugreek exhibited one of the lowest levels of fungal contamination among the analyzed samples (Mir et al., 2024).

## 5 Phytochemical composition of *Trigonella foenum-graecum*

### 5.1 Phytochemical composition of leaves

Recent phytochemical investigations have shown that fenugreek is a rich source of saponins, galactomannans, polyphenols, alkaloids, flavonoids, and stilbene compounds. Fenugreek contains 23%–26% protein, 6%–7% fat and 58% carbohydrates of which about 25% is dietary fiber (Wani and Kumar, 2018).

About 86.1 percent of moisture was found in fenugreek leaves, which also contain carbohydrates, proteins, fiber, fat and minerals at various ratios between 6% and 0.9% (Syed et al., 2020). Shown in Table 1, seven different types of saponins were reported from the leave extract which are commonly known as graecunins (Wani and Kumar, 2018).

**TABLE 1:** Chemical constituents of fenugreek.

S. No	Compound group	Chemical constituents	Plant part	References
1	Alkaloids	Trigonelline	Seed	Srinivasan (2006)
		Choline	Seed	Hassan and Jassim (2018)
		Betaine	Seed	Nagulapalli Venkata et al. (2017)
		Neurin	Seed	Singh et al. (2023)
		Gentianine	Seed	Wani and Kumar (2018)
		Trimethylamine	Seed	Singh et al. (2023)
		Carpaine	Seed	Ahmad et al. (2016)
2	Amino acids	Isoleucine	Seed	Wani and Kumar (2018)
		Lysine	Seed	Bukhari et al. (2008)
		Leucine	Seed	Wani and Kumar (2018)
		Histidine	Seed	Visuvanathan et al. (2022)
		Tyrosine	Seed	Ahmad et al. (2016)
		Arginine	Seed	Vígh et al. (2017)
		Cystine	Seed	Ahmad et al. (2016)
		L-tryptophan	Seed	Bukhari et al. (2008)
		4-hydroxyisoleucine	Seed	Sarker et al. (2024)
3	Saponins	Graecunin	Seed	Vígh et al. (2017)
		Fenugrin B	Seed	Ahmad et al. (2016)
		Fenugreekine	Seed	Singh et al. (2023)
		Trigofoenosides A-G	Seed	Vígh et al. (2017)
4	Steroidal sapogenins	Yamogenin	Seed	Singh et al. (2023)
		Diosgenin	Seed	Navarro Del Hierro et al. (2021)
		Smilagenin	Seed	Hassan and Jassim (2018)
		Sarsasapogenin	Seed	Singh et al. (2023)
		Tigogenin	Seed	Hassan and Jassim (2018)
		Neotigogenin	Seed	Singh et al. (2023)
		Yuccagenin	Seed	Vígh et al. (2017)
		Gitogenine	Seed	SatheeshKumar et al. (2010)
		Neogitogenin	Seed	Vígh et al. (2017)
5	Flavonoids	Quercetin	Leaves	Sambandam et al. (2016)
		Rutin	Seed	Bouhenni et al. (2021)
		Vitexin	Seed	Visuvanathan et al. (2022)
		Isovitexin	Seed	He et al. (2015)
		Isoorientin	Seed	Ahmad et al. (2016)
		Orientin	Seed	Fahmy et al. (2025)
		Luteolin	Seed	Prakash et al. (2025)
		Kaempferol	Leaves	Gikas et al. (2011)
​	​	Tricin-7-O-glucoside	Seed	Vígh et al. (2017)
		Saponaretin	Seed	Singh et al. (2023)
		Genistein	Seed	Vígh et al. (2017)
		Schaftoside	Seed	Vígh et al. (2017)
		Medicarpin	Leaves and stems	Tanimoto et al. (2025)
		Scoparin	Seed	Vígh et al. (2017)
		Naringenin	Leaves, flowers, and stems	Nagulapalli Venkata et al. (2017)
6	Lipids	Triacylglycerols	Seed	Sambandam et al. (2016)
		Diacylglycerols	Seed	Singh et al. (2023)
		Free fatty acids (linoleic acid and alpha-linolenic acid)	Seed	Akbari et al. (2019)
7	Others	Coumarin	Root, pod, stem, and leaves	Vígh et al. (2017)
		Vitamins	Leaves and seeds	Srinivasan (2006)
		Minerals	Leaves and seeds	Nagulapalli Venkata et al. (2017)
		Gum	Seed	Wani and Kumar (2018)

The mineral and vitamins present in the leaves include Zn, Ca, P, Fe carotene, riboflavin, niacin, thiamine and vitamin C (Srinivasan, 2006). The ascorbic acid content in fresh leaves was calculated as 276 mg per 100 g, and leaves are also a good source of Ca, β-carotene and folic acid. Although, it has been reported that about 84.94% and 83.79% ascorbic acid were reduced when put under the sun or oven-dried. A better condition of nutrient retention in the leaves includes storage of leaves at a lower temperature. Better storage conditions include oven-dry and blanching for few minutes (Pal and Mukherjee, 2020).

### 5.2 Phytochemical composition of seeds

Fenugreek seeds are rich in phytochemicals, including alkaloids, flavonoids, and saponins (Singh and Garg, 2006). The seeds contain 5%–10% fixed oils, 20%–30% protein rich in tryptophan and lysine, and 45%–60% carbohydrates mainly as galactomannan fibers. Pyridine alkaloids (choline, trigonelline, carpaine, gentianine), flavonoids (quercetin, orientin, vitexin, isovitexin, luteolin, apigenin) and free amino acids (4-HIL, histidine, lysine, arginine). Hydrolysis of saponin glycosides results in the formation of 0.6%–1.7% steroidal sapogenins, including diosgenin, tigogenin, neotigogenin, and yamogenin, as well as sitosterol, cholesterol and nicotinic acid. Furthermore, about 0.015% of volatile oil is present, mainly represented by n-alkanes and sesquiterpene compounds (Visuvanathan et al., 2022). Fenugreek contains two types of furostanol glycosides, which function as F-ring-opened precursors of diosgenin and are described as hederagenin glycosides. When tissue cultures derived from fenugreek seeds are grown under optimal conditions, they may accumulate up to 2% diosgenin, while trigogenin and gitogenin are present in smaller amounts (Wani and Kumar, 2018). Composition analysis revealed that the endosperm fraction of plant seeds is particularly rich in saponins (4.63%). Moreover, previous studies have suggested that the protein quality of fenugreek exceeds that of many other plant proteins (Pardhe and Jawale, 2022).

Quantitative analyses have reported alkaloid contents of approximately 1.8%, with trigonelline being one of the major alkaloids identified in fenugreek seeds. Studies show that most flavonoids in fenugreek are present as glycosides, where flavonoids are attached to sugars through C- or O-glycosidic bonds. Examples include quercetin-3-O-rhamnoside (quercitrin), vitexin-7-O-glucoside (afroside), and apigenin-6-C-glucoside (isovitexin) (Visuvanathan et al., 2022). Fenugreek seeds also contain several polyphenolic compounds, such as rhaponticin and isovitexin, along with coumarin derivatives and the saponin fenugrin B (He et al., 2015). Furthermore, the seeds are a source of amino acids containing nitrogen, among them glutamic acid, tyrosine, aspartic acid, phenylalanine, and leucine (Nagulapalli Venkata et al., 2017).

Fenugreek seeds contain fiber, gum, various chemical compounds, and volatile constituents. They contain approximately 45% dietary fiber, including about 32% insoluble and 13% soluble fiber (Wani and Kumar, 2018). Fenugreek seeds contain about 50% edible dietary fiber, which is among the highest levels reported for natural fiber sources. Nearly 30% of the seed weight consists soluble fiber, which forms a gel, similar to that of oat bran, psyllium husk, and guar gum (Srinivasan, 2006). The major polysaccharides of fenugreek are galactomannans, composed of a β-(1→4)-linked mannose backbone with α-(1→6)-linked galactose units (Nagulapalli Venkata et al., 2017).

The aerial parts of fenugreek mainly contain Ca, whereas the seeds are rich in K, and P, Mg, Zn, Na, Fe, Cr and Mn are present in moderate amounts (Kaur et al., 2025; Syed et al., 2020). Fenugreek sprouts contain more than twice the Fe level found in fenugreek seeds (Khoja et al., 2021). In addition, the lipid content varies depending on genotype, typically ranging from about 6% to 15%, and consists mainly of triacylglycerols, fatty acids, sterols, and tocopherols (Ciftci et al., 2011).

Fenugreek seeds contain small amounts of volatile and fixed oils (Zemzmi et al., 2020). The fixed oil has a specific gravity of 0.91, an acid value of 1–2 mg KOH/g oil, a saponification value of 178–183 mg KOH/g oil, an iodine value 115 g I_2_/100 g oil, unsaponifiable matter of 3.9%. Its fatty acid composition consisting of palmitic acid (9.6%), stearic acid (4.9%), arachidic acid (2%), oleic acid (35.1%), linoleic acid (33.7%), and α-linolenic acid (13.8%) (Srinivasan, 2006). Volatile oil accounts for about 0.02%, with anethole reported as the major component. The characteristic aroma of fenugreek seeds is attributed to several volatile compounds, including diacetyl, sotolon, eugenol, and others (Kilambi and Shah, 2021). Out of all these volatile compounds, sotolon was reportedly the most predominant in 95% (5s)-enantiomeric form. After fenugreek consumption can lead to a maple-syrup smell, mainly caused by the compound 2,5-dimethylpyrazine (Wani and Kumar, 2018).

Limited information is obtained on the stem bark of fenugreek. Approximately 28% mucilage is present, along with volatile oil, nicotinic acid, trigocoumarin, and trigonelline, as well as 5% strongly scented bitter fixed oil, 22% proteins, and a yellow coloring compound (Wani and Kumar, 2018).

## 6 Pharmacological profiles and effects of fenugreek

### 6.1 Antioxidant effect

Excessive formation of reactive oxygen species (ROS) leads to oxidative damage to proteins and lipids. These injuries are associated with the development of chronic degenerative diseases (Visuvanathan et al., 2022). The results of several studies indicate that fenugreek has potential antioxidant properties. However, reported antioxidant capacity can vary widely, depending on the extraction solvent, germination conditions, phytochemical composition, and experimental assay used. Methanol and ethanol extracts of fenugreek seeds exhibit the highest 2,2-diphenyl-1-picrylhydrazyl (DPPH) radical-scavenging activity due to the greater extraction of phenolic compounds by these highly polar solvents (Bukhari et al., 2008). Fenugreek seed oil showed strong antioxidant activity, with ABTS exhibiting greater radical-scavenging capacity than DPPH assay, with half maximal inhibitory concentration (IC_50_) values of 161.3 and 172.6 μg/mL, respectively (Akbari et al., 2019). In general, polar extracts tended to show stronger antioxidant activity, most likely because they contained higher levels of phenolics and flavonoids. Fenugreek seed extracts demonstrated increased oxidative stability in duck meat proteins, scavenging 58.79% of hydroxyl radicals and reducing protein oxidation, as evidenced by a 42.0% reduction in carbonyl formation and a pronounced decrease in sulfhydryl loss (Chen et al., 2023). This implies that compounds obtained from fenugreek play a role not only in scavenging free radicals but can also prevent the formation of oxidized proteins.

**FIGURE 2:**
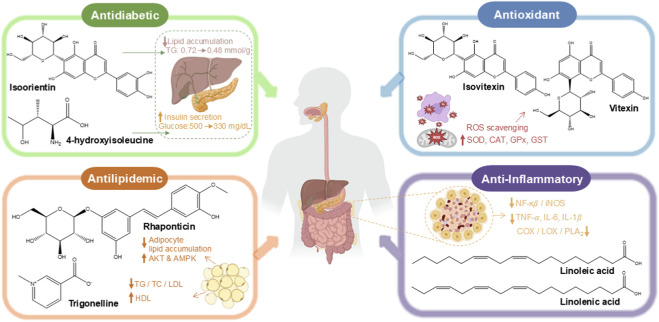
Systemic metabolic effects of fenugreek bioactive constituents.

Germinated fenugreek seeds showed higher phenolic content (39.2 mg GAE/g) and flavonoid content (27.8 mg QE/g) (Khole et al., 2014). These findings indicate that germinated seeds may offer a more effective option for diabetes management compared to ungerminated seeds. These findings suggest that germination may enhance fenugreek’s antioxidant potential by promoting a greater accumulation of phenolic and flavonoid compounds. Vitexin and isovitexin were found to be the main antioxidant constituents, the structures of which are shown in Figure 2 (Peng et al., 2008). In addition, the ethanolic extract of fenugreek also demonstrated clear free-radical-scavenging activity *in vitro* (Sekhar et al., 2024). Recent investigations have also focused on using fenugreek-based materials for defense against oxidative damage. Pre-treatment with the fenugreek extract and bovine serum albumin (BSA) composite soft hydrogel significantly increased cell viability after UVB exposure, with more than 80% of cells remaining viable after 20 min, while the fenugreek extract-BSA film preserved viability above 85% (Tripathi et al., 2026). This protective effect was attributed to the high content of polyphenols, flavonoids, and polysaccharides in fenugreek extract, which neutralize UVB ROS and limit oxidative cellular damage (Abdennebi et al., 2025).

*In vivo* studies have also demonstrated the antioxidant potential of fenugreek in both animal and plant stress models. The ethyl acetate extract of fenugreek seeds showed the strongest biological effect in cholesterol-fed rats by reducing thiobarbituric acid-reactive substances (TBARS) levels and enhancing antioxidant enzyme activity. In the same study, methanol and ethyl acetate extracts exhibited the highest phenolic and flavonoid contents (Belguith-Hadriche et al., 2013). Fenugreek seed powder reduced lipid peroxidation and increased hepatic antioxidant enzyme activities of glutathione peroxidase (GPx), superoxide dismutase (SOD), glutathione-S-transferase (GST), and catalase (CAT), showing attenuation of oxidative stress associated with inflammation (Yadav and Baquer, 2014). Trigonelline from fenugreek restored antioxidant enzyme activities and reduced malondialdehyde (MDA) levels in alcohol-intoxicated rats (Sekhar et al., 2024).

Apart from animal studies, several investigations have examined the antioxidant defense responses of fenugreek under environmental stress conditions. Fenugreek exposed to nickel stress activated antioxidant defense systems, including SOD, CAT, and peroxidase (POX), as well as changes in hydrogen peroxide (H_2_O_2_), MDA, proline (PRO), while exogenous calcium further enhanced these antioxidant responses (Aqeel et al., 2022). Furthermore, the combined application of biofertilizer and acidified carbon (1.50 CANBC + BF) improved fenugreek quality by reducing MDA levels and electrolyte leakage (EL) while increasing chlorophyll content (Qian et al., 2023). Silicon supplementation in salt-stressed fenugreek significantly reduced MDA and H_2_O_2_ levels while increasing the activities of SOD, CAT and guaiacol peroxidase (Lamsaadi et al., 2023). It was also demonstrated that UVB exposure elevated oxidative stress in fenugreek by increasing MDA and electrolyte leakage. However, melatonin and ascorbic acid treatments reduced these effects and enhanced enzymatic and non-enzymatic antioxidant responses under UVB stress (Hajipour et al., 2025).

Human clinical evidence regarding the antioxidant activity of fenugreek remains limited. In a parallel-group randomized clinical trial involving 48 patients with type 2 diabetes mellitus, supplementation with 15 g/day fenugreek seed powder for 8 weeks increased SOD activity from 19.80 to 31.90 U/mL (p = 0.001). In contrast, no significant between-group effects were observed for total antioxidant capacity (TAC) (p = 0.72) and GPx activity (p = 0.47). However, no significant effects were found for Interleukin-6 (IL-6) or Tumor Necrosis Factor-alpha (TNF- α ). The study included only 48 participants and an 8-week intervention period, and the absence of placebo blinding may have increased the risk of bias and limited the generalizability of the results (Tavakoly et al., 2018).

Current evidence demonstrates that fenugreek exhibits substantial antioxidant activity *in vitro*, *in vivo*, and under environmental stress conditions. The antioxidant efficacy appears to depend strongly on extraction method, germination status, and phytochemical composition, with phenolic- and flavonoid-rich extracts generally exhibiting the strongest activity. However, despite promising preclinical findings, further well-designed clinical studies are still required to confirm the therapeutic relevance of these antioxidant effects in humans.

### 6.2 Antidiabetic effect

Diabetes is a common global health disorder with significant social and economic effects. The disease results from insulin deficiency in type 1 diabetes and insulin resistance in type 2 diabetes (Subramanian and Prasath, 2014). Fenugreek seeds have long been traditionally known for their antidiabetic action (Singh et al., 2025c). The therapeutic role of *T. foenum-graecum* in type 1 and type 2 diabetes are associated with the normalization of enzyme activities involved in glucose and lipid metabolism to normal levels (Pradeepkiran et al., 2020).

Several bioactive compounds found in fenugreek seeds, such as saponin, 4-HIL, galactomannan, quercetin, diosgenin, vitexin, isovitexin, and trigonelline are known to help reduce hyperglycemia, as illustrated in Figure 2 (Sarker et al., 2024). Among these compounds, galactomannan is considered the main active compound in fenugreek and may be useful as a potential treatment for diabetes. In streptozotocin (STZ)-induced diabetic rats treated with fenugreek galactomannan (0.5 g/kg/day) for 28 days, blood glucose levels decreased from 20.4 ± 3.2 to 17.8 ± 2.3 mM, whereas untreated diabetic rats maintained glucose levels around 21.2 ± 2.0 mM (Jiang et al., 2017). The saponin and sapogenin fractions exhibit strong α-glucosidase inhibitory activity, with sapogenins, formed by saponin hydrolysis, showing stronger inhibition than saponins due to the absence of sugar chains. Notably, the inhibitory activity against α -glucosidase varied among the extracts, with IC_50_ values ranging from 5.49 to 15.16 μ M (Zhang et al., 2020). Similarly, four flavonoid glycosides from fenugreek seeds, namely, isoorientin, vitexin, orientin, and isovitexin were investigated. The most active among of them was isoorientin, which reduced intracellular triglyceride content from approximately 0.72 to 0.48 mmol/g protein in differentiated 3T3-L1 adipocytes, reduced lipid accumulation by downregulating peroxisome proliferator activated receptor γ (PPARγ), CCAAT/enhancer binding protein (C/EBPα) and fatty acid synthase (FAS), restored insulin-stimulated glucose uptake protein kinase B (AKT) and AMP-activated protein kinase (AMPK) (Luan et al., 2018).

Ethanol fractions of *Zingiber officinale* exhibited strong antidiabetic activity, including 95% antiglycation and 54.97% α-amylase inhibition, whereas *T. foenum-graecum* leaf extracts showed lower α-amylase inhibition ranging from 9.43% to 24.95% (Hafeez et al., 2023). In contrast, compared with leaf extracts, germinated fenugreek preparations demonstrated substantially stronger enzyme inhibitory activity. Germination enhanced the antidiabetic potential of fenugreek galactomannan by its strong α-glucosidase inhibition as 86.89%, demonstrating improved glucose regulation (Zhu et al., 2024). Similarly, germinated fenugreek sprout extract IM6E, obtained from seeds of ten different fenugreek genotypes and enriched in quercetin (0.148%), present at higher levels than diosgenin and trigonelline, exhibited strong α-glucosidase inhibitory activity (95.24%) (Laila et al., 2023). Taken together, these studies demonstrate that germination and phytochemical enrichment can significantly enhance the antidiabetic properties of fenugreek.

The antidiabetic effects of fenugreek have been observed in diabetic rats (Arshadi et al., 2015; Bafadam et al., 2021; Baliga et al., 2017; Khan et al., 2012; Kumar et al., 2005; Laila et al., 2023; Pradeepkiran et al., 2020), in diabetic mice (Alluri et al., 2020; Alsieni et al., 2021; Eidi et al., 2007; Li et al., 2024; Suresh Babu and Srinivasan, 1998; Uemura et al., 2011), in diabetic rabbits (Alluri et al., 2020; Khan et al., 2012; Puri et al., 2011; Sarker et al., 2024) and in diabetic dogs (Alluri et al., 2020; Eidi et al., 2007; Khan et al., 2012; Sarker et al., 2024; Srinivasan, 2006).

Several bioactive constituents of fenugreek have demonstrated significant beneficial effects on glucose and lipid metabolism in diabetic models. For example, 4-HIL isolated from seeds of fenugreek lowered plasma glucose levels from approximately 500 to 330 mg/dL after 4 weeks of treatment (50 mg/kg/day) in STZ-induced diabetic rats, demonstrating strong insulin-independent antidiabetic activity in both type 1 and type 2 diabetes models. Mechanistically, 4-HIL enhances glucose-dependent insulin secretion and improves insulin sensitivity, thereby contributing to glucose homeostasis (Haeri et al., 2012). Similarly, diosgenin reduced hepatic triglycerides (TG) from 12.4 ± 0.86 to 6.07 ± 0.74 mmol/g after 4 weeks of treatment with 2% fenugreek supplementation, and lipogenic gene expression in diabetic KK-Ay mice by inhibiting TG accumulation and liver-X-receptor-α (LXRα) activity, thereby improving dyslipidemia (Uemura et al., 2011). Comparable findings were observed for trigonelline, which reduced blood glucose levels in both diabetic rats (Subramanian and Prasath, 2014) and humans (Gupta et al., 2021), while also enhancing metabolic status and restoring normal lipid levels in high-fat-diet (HFD)-fed/STZ-induced type 2 diabetic rats (Subramanian and Prasath, 2014).

In addition to glucose lowering, several studies demonstrated improvements in pancreatic, hepatic, and renal function. Fenugreek flavonoids showed antidiabetic effects in STZ-induced rats by lowering fasting blood glucose and improving islet and kidney status. Additionally, *T. foenum-graecum* F-GAL produces antidiabetic effects in STZ-induced rats, whereas after 28 days of treatment, 14 metabolic biomarkers associated with improvements in tryptophan, histidine, phenylalanine, glycerophospholipid, sphingolipid, and arachidonic acid metabolism were identified, indicating that F-GAL acts through multiple metabolic pathways involved in diabetes (Jiang et al., 2017). Similarly, alkaloid extracts of fenugreek seeds significantly increased serum insulin levels, repaired liver, lowered blood glucose in STZ-induced diabetic rats (Helmy et al., 2007).

Comparable protective effects were also reported for different fenugreek preparations. Ethanolic extracts of fenugreek significantly lowered serum glucose, urea, uric acid, aminotransferase (AST) and alanine aminotransferase (ALT), and prevented weight loss in STZ-induced diabetic rats (Eidi et al., 2007), whereas seed mucilage produced a 26% decrease in fasting glucose and a 30% improvement in urine sugar and volume, exceeding turmeric, which achieved only an 18% glucose reduction (Kumar et al., 2005).

Spices represent an important group of dietary adjuncts, many of which possess diverse physiological and pharmacological activities in addition to improving the taste and flavor of foods. Dietary fenugreek seeds (100 g/kg) combined with onion *Allium cepa* L. (30 g/kg) improved heamatological indices and erythrocyte membrane lipid profile, reducing osmotic fragility, lipid peroxidation in STZ-induced diabetic rats (Pradeep and Srinivasan, 2018). Likewise, aqueous extracts of fenugreek and *Nigella sativa* seeds, improved glucose, insulin levels and pancreatic β-cell number in alloxan-induced type 2 diabetic rats (Mohamed et al., 2015). Consistent with these findings, IM6E significantly reduced post-prandial hyperglycemia and improved glucose tolerance in normal and STZ-induced diabetic rats (Laila et al., 2023). Similarly, the water-soluble compound GII purified from fenugreek seeds improved diabetic metabolism in alloxan-induced rabbits. It decreased elevated serum lipids, increased liver and muscle glycogen content and repaired histopathological damage in the pancreas, liver, heart and kidneys (Puri et al., 2011).

Differences in antidiabetic activity among fenugreek-derived extracts suggest that germinated and phytochemical-enriched extracts exhibit stronger biological effects than conventional leaf or crude extracts, likely due to their higher content of bioactive compounds such as quercetin, diosgenin, trigonelline, and 4-HIL. According to this, beneficial effects observed across different diabetic animal models highlight the broad therapeutic potential of fenugreek in the management of metabolic disorders.

Human clinical studies also support the antidiabetic potential of *T. foenum-graecum*. In a 12-week randomized single-blind placebo-controlled clinical study involving 64 newly diagnosed type 2 diabetic patients, supplementation with 1 g/day fenugreek galactomannan significantly reduced fasting blood glucose levels from 7.75 to 6.30 mmol/L. Moreover, significant reduction in total cholesterol (TC) (5.15–4.57 mmol/L), triglycerides (2.50–1.88 mmol/L), and low-density lipoprotein (LDL) (2.65–1.89 mmol/L) levels were also observed (Rashid et al., 2019). However, the study included only 64 participants, employed a single-blind design, and evaluated a single galactomannan dose (1 g/day) over a 12-week period, which may limit the generalizability of the findings. Similarly, in another randomized clinical study involving patients with type 2 diabetes mellitus, fenugreek seed powder supplementation for 8 weeks significantly reduced fasting blood glucose levels from 151.33 to 112.33 mg/dL. In addition, significant reduction in triglyceride and LDL levels were also observed, while high-density lipoprotein (HDL) levels significantly increased after treatment (Wankhede et al., 2016). However, the effects of fenugreek on HDL levels remain inconsistent across clinical studies. Furthermore, pharmacokinetic enhancement suggests that fenugreek taken together with metformin help maintain lower blood glucose levels more effectively than metformin alone (Abdelwahab et al., 2021).

### 6.3 Antilipidemic effect

In recent years, *T. foenum-graecum* has attracted increasing interest as a potential dietary agent for the improving of blood lipid levels, reflecting the growing interest in plant-based alternatives to conventional hypolipidemic therapy. Previous pharmacological studies have indicated that fenugreek seed extracts exhibit antihyperglycemic and antihyperlipidemic effects (Hinad et al., 2024).

*Trigonella foenum-graecum* is a source of phenolic compounds, including rhaponticin (Figure 2), which are associated with antioxidant activity and improvement of insulin sensitivity. These mechanisms may indirectly contribute to the regulation of lipid metabolism (Singh and Yadav, 2022). Consistent with these observations, polyphenolic stilbenes isolated from *T. foenum-graecum* seeds reduced lipid accumulation and intracellular triglyceride levels in insulin-resistant 3T3-L1 adipocytes at 10 μ mol/L. This effect was associated with suppression of adipocyte-specific proteins and activation of AKT and AMPK signaling (Li et al., 2018).

Supplementation with *T. foenum-graecum* powder (8 g/100g diet) for 4 weeks reduced serum TG by 34.2%, TC by 40.7%, low-density lipoprotein cholesterol (LDL-c) by 71.1% and very low-density lipoprotein cholesterol (VLDL-c) by 32%, while increasing HDL-c levels by 6.5% in hamsters fed a high-cholesterol diet (HCD). The study included 32 male hamsters (n = 8/group) and additionally demonstrated a 7.8-fold increase in hepatic hepatic low-density lipoprotein receptor (LDLR) gene expression in the HCD + FG8 group, whereas the lower dose (2 g/100 g diet) showed no significant effects on lipid profile (Kassaee et al., 2021). Similarly, trigonelline treatment (150 mg/kg for 30 days) reduced serum cholesterol by approximately 31%, TG by 26%, and free fatty acid levels by 32% in HFD and STZ-induced diabetic rats. However, the study was limited by a relatively small sample size (n = 6/group) (Subramanian and Prasath, 2014). Consistent with these findings, treatment with saponin isolated from fenugreek seeds (6–24 mg/kg for 9 weeks) improved lipid profiles in HFD-fed rats by reducing TC from 1.83 to 1.50–1.53 mmol/L and TG from 0.76 to 0.57–0.62 mmol/L, mainly through modulation of cholesterol metabolism rather instead of suppression of intestinal cholesterol absorption (Chen et al., 2017).

In HFD-fed C57BL/6J mice, fenugreek supplementation improved the HDL/LDL ratio and reduced LDL cholesterol levels, while no significant changes in TC, TG, body weight or adiposity were observed (Knott et al., 2017). In contrast, *T. foenum-graecum* seed extracts were also reported to suppress lipid peroxidation and to improve metabolic abnormalities associated with diabetes, including dyslipidemia (Chakraborty et al., 2016). These effects may partly be related to modulation of hepatic lipid accumulation, since the observed improvements in plasma lipid profile were associated with altered hepatic lipid accumulation, decreased liver FABP4/aP2 expression and increased adiponectin levels (Knott et al., 2017).

Saponins were identified as major bioactive compounds of fenugreek seeds responsible for the reported hypolipidemic activity in the experimental dyslipidemia model (Chen et al., 2017). Similarly, trigonelline improved insulin sensitivity, enhancing insulin-stimulated glucose uptake, and normalizing lipid metabolism, resulting in reduced serum cholesterol, triglyceride, and free fatty acid levels (Subramanian and Prasath, 2014). In addition, flavonoids isolated from ethyl acetate seed extracts exhibited hypocholesterolemic activity, while antioxidant constituents from the seeds were suggested to contribute to the control of elevated blood cholesterol levels (Ahmad et al., 2016). Furthermore, 4-HIL isolated from the plant was reported to enhance insulin sensitivity and reduce hyperlipidemia (Haeri et al., 2012).

In a randomized double-blind placebo-controlled trial involving 48 prediabetic adults, daily supplementation with 500 mg of fenugreek seed extract for 12 weeks maintained TG levels stable (from 1.62 to 1.58 mmol/L), whereas TG levels increased in the placebo group (from 1.97 to 2.28 mmol/L). However, no significant changes were observed in total TC, HDL, LDL, or body mass index (BMI) between groups (Pickering et al., 2022). Similarly, a meta-analysis of 12 randomized controlled trials involving 560 participants showed that fenugreek supplementation significantly lowered TC, TG, and LDL levels, increased HDL cholesterol in adults, and had no effect on BMI (Askarpour et al., 2020). Fenugreek seed powder positively affected lipid metabolism in type 2 diabetic patients by reducing TC by about 14%, TG by around 24%, and LDL by about 23%, while increasing HDL by nearly 22% (Geberemeskel et al., 2019). In this context, accumulating preclinical and mechanistic studies indicate that *T. foenum-graecum* may influence metabolic homeostasis through pathways related to lipid metabolism and oxidative stress (Choudhury et al., 2018). No significant effect on BMI was observed, suggesting that the beneficial effects of fenugreek may be more directly related to modulation of lipid profile rather than an effect mediated by changes in body weight.

In experimental and animal studies, *T. foenum-graecum* showed significant hypolipidemic effects, including reductions in TG, TC, and LDL levels through regulation of lipid metabolism and insulin-related pathways. In contrast, clinical studies demonstrated more moderate but consistent improvements in lipid profile, mainly reflected by decreased or stabilized TG and LDL levels without significant changes in BMI. Collectively, the available evidence indicates that *T. foenum-graecum* possesses promising lipid-lowering potential, primarily associated with modulation of lipid metabolism and insulin sensitivity rather than body weight reduction.

### 6.4 Anti-inflammatory effect

Fenugreek exhibits significant anti-inflammatory activity in various *in vitro* models through modulation of inflammatory mediators, cytokine production, and enzyme activity. Seed extracts inhibited nitric oxide production in lipopolysaccharide (LPS)-activated human monocytic cell line (THP-1)-derived macrophages in a dose-dependent manner at low concentrations and reduced the secretion of pro-inflammatory cytokines, including TNF-α and IL-6 (Kmail et al., 2022). Consistently, the same study reported suppression of LPS-induced NF-κB activation and inducible nitric oxide synthase (iNOS) expression, resulting in lower nitric oxide levels in macrophages (Kmail et al., 2022). Furthermore, flavonoid- and tannin-rich aqueous and ethanolic *T. foenum-graecum* extracts reduced IL-6 and TNF-α production (Kmail et al., 2022). Similarly, a steroidal glycoside-rich extract prepared from fenugreek seeds reduced pro-inflammatory cytokine production in macrophages and downregulated TNF, IL-6, IL-1β and NF-κB gene expression (Sen et al., 2025). In addition, fenugreek inhibited the inactive rhomboid protein two (iRhom2)/TNF-α converting enzyme (TACE) axis, leading to reduced TNF-α release from adipocytes (Zhou et al., 2020).

The anti-inflammatory effects of fenugreek are closely associated with its rich content of bioactive constituents such as alkaloids, saponins, and flavonoids, which regulate the function of inflammatory cells such as mast cells, macrophages, lymphocytes, as well as neutrophils. Mechanistically, these effects are partly associated with inhibition of phospholipase A2 (PLA2), cyclooxygenase (COX), and lipoxygenase (LOX) (Cheurfa et al., 2022). In agreement with these findings, fenugreek extracts showed pronounced anti-inflammatory activity by inhibiting LOX, exhibiting IC_50_ values around 19.69 μg/mL (Neagu et al., 2023). Fenugreek also significantly suppressed LOX and hyaluronidase (HYA) with the IC_50_ value of 19.69 μg/mL and 17.57 μg/mL (Christella et al., 2025). Moreover, phenolic acids and flavonoids present in fenugreek extracts were associated with inhibition of HYA inhibition (Neagu et al., 2023).

The seeds and leaves of *T. foenum-graecum* show anti-inflammatory effects in animal and human models. At a dose of 200 mg/kg, aqueous-ethanolic and aqueous seed extracts produced substantial suppression of carrageenan-induced inflammation, reaching 63.23% and 46.77%, respectively (Cheurfa et al., 2022). Consistent with previous findings, extracts of fenugreek significantly suppressed carrageenan-induced paw oedema in rats (Pournamdari et al., 2018). Similarly, *in vivo* treatment led to a gradual reduction of paw oedema within 1–6 h, with maximal inhibition of around 63% at doses of 100 and 200 mg/kg (Shaukat et al., 2023). Petroleum-ether and hydroethanolic seed extracts lowered paw edema by up to 85%, decreased circulating IL-6, Interleukin-1β (IL-1β) and TNF-α (Christella et al., 2025). Comparable anti-inflammatory effects were observed with petroleum ether extract from of fenugreek seeds reduced paw inflammation leading to a 37% decrease in carrageenan-induced edema and by 85% in the formaldehyde-induced edema model (Pundarikakshudu et al., 2016).

In addition to reducing oedema, fenugreek extracts modulated inflammatory and oxidative stress markers *in vivo*. Hydroethanolic seed extracts of *T. foenum-graecum* markedly reduced cellular infiltration at inflammatory sites in air-pouch inflammation and peritonitis induced by carrageenan models and significantly improved SOD, CAT, MDA and myeloperoxidase (MPO) (Fatima et al., 2022). Furthermore, flavonoids were reported to inhibit inflammatory mediator synthesis and significantly reduce the serum COX-2-mediated prostaglandin E2 (PGE2) levels (Shaukat et al., 2023). The anti-inflammatory activity was also associated with linolenic- and linoleic-acid-rich fractions derived from fenugreek seeds and were accompanied by reductions in serum glutamate-pyruvate transaminase (SGPT) and alkaline phosphatase (ALP) activities (Pundarikakshudu et al., 2016) (Figure 2).

Fenugreek also demonstrated beneficial anti-inflammatory effects in metabolic disorders. Oral administration of a steroidal glycoside-rich fenugreek extract significantly reduced serum TNF-α, IL-6 and IL-1β while increasing the anti-inflammatory cytokines IL-10 and IL-4 in obese mice (Sen et al., 2025). Moreover, the anti-inflammatory and antioxidant properties of fenugreek help prevent glucose intolerance and dyslipidemia in HFD-fed rats (Jahan et al., 2024). Reductions in systematic inflammation were accompanied by improved glucose tolerance, insulin sensitivity and homeostasis model assessment of insulin resistance (HOMA-IR) values. This indicates a close link between anti-inflammatory and metabolic effects (Sen et al., 2025).

Human clinical studies also suggest the anti-inflammatory potential of *T. foenum-graecum.* In a double-blind randomized placebo-controlled trial involving 101 women with primary dysmenorrhea, administration of 900 mg fenugreek seed powder three times daily for two menstrual cycles significantly reduced pain severity from 6.4 to 3.25, decreased pain duration, and vomiting compared with placebo (Akhtari et al., 2024). In another randomized double-blind placebo-controlled study, fenugreek hydro-ethanolic extract supplementation (250 mg twice daily for 42 days) reduced leg and joint pain, hot flashes, and night sweats in postmenopausal women (Thomas et al., 2020).

*Trigonella foenum-graecum* demonstrated considerable anti-inflammatory potential in both experimental and clinical studies. Its beneficial effects were mainly associated with suppression of inflammatory mediators, regulation of NF-κB pathways, and reduction of inflammation-associated symptoms, supporting the therapeutic relevance of fenugreek as a natural anti-inflammatory agent.

### 6.5 Antibacterial and antifungal effect

Essential oil extracted from *T. foenum-graecum* seeds powder exhibited antibacterial activity against *Proteus vulgaris (P. vulgaris)*, *Paracoccus denitrificans (P. denitrificans)*, *Escherichia coli*, *X. campestris (Xanthomonas campestris)*, *Bacillus subtilis*, *Sarcina lutea (S. lutea)* and *Klebsiella pneumoniae*, producing inhibition zones ranging from 8 to 15 mm (Moniruzzaman et al., 2015). Fenugreek seed mucilage (FSM) was extracted and subsequently used to develop active edible films incorporating carboxymethyl cellulose and rosemary essential oil (REO), demonstrating its potential as an antimicrobial agent. Biological assays showed that REO-loaded biofilms exhibited antimicrobial activity against *S. aureus* and *E. coli* (Lindi et al., 2024). Ethyl acetate extracts of fenugreek microgreens exhibited antibacterial activity against *Aeromonas hydrophila (A. hydrophila)*, *Pseudomonas aeruginosa* and *S. aureus* with inhibition zones up to 24.4 mm (Jayaraman and Ramasamy, 2024). Fenugreek seeds irradiated with gamma rays yielded the bioactive compound 4-HIL, which inhibited *M. oryzae (Magnaporthe oryzae)* and reduced fungal growth by up to 69.9% *in vitro* (Gajbar et al., 2022). Fenugreek seed dye displayed effective antimicrobial activity against pathogenic microbes (Selvam et al., 2015).

The antimicrobial mechanisms and anticancer activity of silver nanoparticles synthesized using the seed extract of *T. foenum-graecum* L. were investigated. The lowest minimum inhibitory concentration (MIC) of the silver nanoparticles (AgNPs) against *Staphylococcus aureus (S. aureus)* was 62.5 μg mL^-1^, while MIC values against *E. coli (E. coli)* and *K. pneumoniae (K. pneumoniae)* were 125 and 250 μg mL^-1^, respectively (Varghese et al., 2019). In a similar study, AgNPs synthesized using fenugreek leaf extract inhibited *S. aureus, E. coli, P. aeruginosa (P. aeruginosa)* and *V. cholerae (Vibrio cholerae)*, with maximum inhibition zones of 12 mm for *E. coli* and 11 mm for *P. aeruginosa* (Ghoshal and Singh, 2022). The combination of Fe_3_O_4_ nanocomposites with fenugreek seed gum and AgNPs showed antibacterial effects against different bacterial strains (Allafchian et al., 2023). Similarly, Ag-ZnO nanocomposites (extract of fenugreek leaves at 20 mg/mL) exhibited antibacterial and antifungal activity against *S. aureus*, *E. coli* and *Candida albicans (C. albicans)* fungi (Noohpisheh et al., 2020). Copper nanoparticles (CuNPs) exhibited strong antimicrobial activity against *K. pneumoniae*, *S. aureus* and *C. albicans* with inhibition zones of about 15–16 mm (El-Batal et al., 2018). SnO_2_ nanoparticles synthesized using seed extract showed antibacterial activity against *B. subtilis (B. subtilis)* and *E. coli* bacteria (Goyal et al., 2022). Similar antibacterial effects were observed for nickel oxide nanoparticles prepared with fenugreek seed extract against *Staphylococcus saprophyticus (S. saprophyticus)* and *E. coli*, with inhibition zones increasing at higher concentrations (Malathi et al., 2023). TiO_2_ nanoparticles synthesized from *T. foenum-graecum* leaf extract demonstrated antibacterial effects against Gram-positive and Gram-negative bacteria, producing inhibition zones ranging from 8.5 to 11.6 mm (Subhapriya and Gomathipriya, 2018).

AgNPs prepared using *T. foenum-graecum* seed extract were examined for antifungal activity. Inhibition of *Aspergillus flavus (A. flavus)*, *Trichophyton rubrum (T. rubrum)* and *Trichoderma viride (T. viride)* was observed with MIC values of 250 μg mL^-1^ (Varghese et al., 2019). Selenium-chitosan nanocomposites prepared using FSM were tested against green mold *Penicillium digitatum (P. digitatum)* on lemons. The nanocomposites produced inhibition zones up to 32.2 mm, showed fungicidal activity at 12.5 mg mL^-1^, and completely suppressed green mold development on coated fruits within 10 days (Tayel et al., 2024). Encapsulation of fenugreek essential oil in CuO nanoparticles strongly inhibited the growth of *Colletotrichum nymphaeae (C. nymphaeae)*, achieving more than 90% mycelial inhibition at higher concentrations (Weisany et al., 2024). Chitosan-assisted fenugreek nanocomposites incorporated into denture soft-lining materials markedly inhibited *C. albicans* proliferation under *in vitro* conditions, with the 2.5% preparation exhibiting the lowest fungal count of 521 (CFU/mL) (Damle et al., 2025).

**FIGURE 3:**
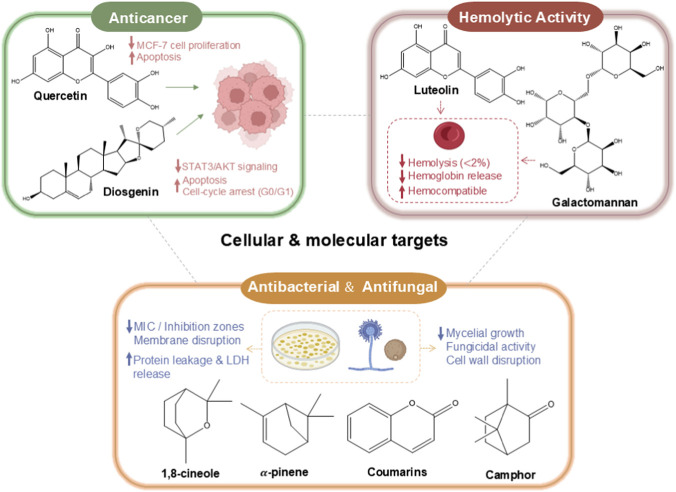
Cellular and molecular targets of major bioactive compounds from *Trigonella foenum-graecum*.

The antimicrobial activity of fenugreek-derived extracts and nanomaterials has been associated with several common mechanisms, including disruption of microbial cell membranes, leakage of intracellular contents, generation of reactive oxygen species (ROS), release of metal ions, and interactions with proteins and nucleic acids. Treatment with AgNPs prepared with the seed extract of *T. foenum-graecum* resulted in increased protein leakage along with elevated lactate dehydrogenase and alkaline phosphatase activities, indicating damage to bacterial cell walls and membranes (Varghese et al., 2019). Related studies reported that plant-mediated AgNPs may act by attaching to bacterial surfaces, leading to membrane disruption and damage to intracellular organelles (Ghoshal and Singh, 2022). The antibacterial activity of SnO_2_ nanoparticles synthesized using seed extract was associated with several mechanisms, including generation of Sn^4+^ ions, ROS production, electrostatic interactions with bacterial cell membranes, nanoparticle penetration into cells, and DNA damage (Goyal et al., 2022). Selenium-chitosan nanocomposites prepared using FSM have shown antimicrobial activity, which is attributed to fenugreek bioactive constituents such as alkaloids, saponins and flavonoids, and vitamins, including trigonelline, fenugreekine, and coumarins, as illustrated in Figure 3 (Tayel et al., 2024).

The antibacterial effect of fenugreek essential oil was attributed to phenolic constituents such as α-pinene, camphor, and 1,8-cineole. Because of their hydrophobic nature, these compounds penetrate lipid molecules within bacterial and mitochondrial membranes, causing enhanced membrane degradation (Lindi et al., 2024). The antimicrobial activity of essential oil extracted from fenugreek seed powder was linked to the combined effects of alkaloids, tannins, flavonoids, terpenoids and phenolic compounds (Moniruzzaman et al., 2015).

The antibacterial and antifungal properties of fenugreek have supported diverse applications, including biomedical, pharmaceutical, cosmetic, food packaging, water treatment, textile fields. Coatings based on FSM containing REO showed potential as sustainable active packaging materials and extended apple shelf life to 30 days (Lindi et al., 2024). Similarly, selenium-chitosan nanoparticles may also be used as edible coatings for lemons to control green mold and preserve fruit quality (Tayel et al., 2024). Fe_3_O_4_/Ag nanocomposites coated with fenugreek seed gum could be applied in water purification because they show antibacterial activity and can be magnetically removed (Allafchian et al., 2023). Recent multi-omics approaches are increasingly being applied to fenugreek to investigate its key bioactive constituents and to support future antimicrobial activity (Kachhwaha et al., 2024). In addition, nano-encapsulation of fenugreek essential oils has been shown to improve antifungal activity (Weisany et al., 2024).

*Trigonella foenum-graecum* demonstrated broad antimicrobial activity against different bacterial and fungal strains in various experimental studies. Its extracts, essential oils, and nanoparticle-based formulations acted mainly through disruption of microbial membranes, induction of oxidative stress, and damage to intracellular components, indicating good potential as a natural antimicrobial agent.

### 6.6 Anticancer effect

Cancer is a major global health challenge affecting millions of people worldwide. Conventional treatments often provide only limited survival benefits and often cause significant side effects. Because of this, alternative medicine increasingly turns into natural products for cancer prevention. Current research shows that fenugreek seeds provide protective effects in various experimental cancer models.

Early *in vitro* studies demonstrated the antiproliferative potential of fenugreek against breast cancer cells. The proliferation of MCF-7 human breast cancer cells was assessed after treatment with soybean and fenugreek extracts (Sebastian and Thampan, 2007). In MCF-7 cells, treatment with aqueous and ethanol soybean extracts stimulated cellular proliferation and enhanced DNA synthesis. In contrast, exposure to ethanol-derived fenugreek extracts reduced cell viability, triggered early apoptotic changes, and produced DNA fragmentation of approximately 180–200 base pairs (Amin et al., 2005). Similarly, fenugreek was identified among 50 spices and herbs as a strong inhibitor of cathepsin G. It suppressed cathepsin G-induced MCF-7 cell aggregation (Li et al., 2010). Both quercetin and trigonelline showed similar activity, suggesting a role in preventing malignant progression of human breast cancer cells induced by cathepsin G (Figure 3).

Recent research findings indicate that fenugreek seed extracts effectively exhibited time- and dose-dependent suppression of pancreatic cancer cell proliferation without significantly affecting normal cells (Morshidi et al., 2025). Likewise, the extract was also found to inhibit the proliferation of breast, prostate, and pancreatic cancer cell lines, induced cell death, downregulated mutant p53 in DU-145 cell, and reduce AKT phosphorylation in PC-3 cell (Hao et al., 2022). Fenugreek extract was found to exert selective cytotoxic effects *in vitro* on different cancer cell lines, including T-cell lymphome, while sparing normal cells. Extracts at 100–300 μg/mL reduced cancer cell viability across all tested time points and proteomics analysis confirmed distinct protein profiles among fenugreek samples from different regions (Alsemari et al., 2014). Moreover, after 72 h of incubation at 400 μg/mL, the seed extract reduced the proliferation of MCF-7 cells, but showed no anticancer activity in liver and Vero cell lines (Al-Timimi, 2019).

A phytochemical and docking study identified 72 fenugreek seed compounds with chemical absorption, distribution, metabolism, excretion, and toxicity (ADMET) properties, with saponins and flavonoids showing strong multitarget binding to cancer-associated proteins (Adhikary et al., 2025). Ultrasound-assisted extraction demonstrated that methanol yielded the highest levels of antioxidant, phenolic, flavonoid and saponin content. Another computational study identified fenugreek seed phytocompounds such as tannins, saponins, steroids as potential AKT-1 inhibitors in cancer (Ahmad et al., 2022). In addition, a study in hepatocellular carcinoma showed that diosgenin blocks signal transducer and activator of transcription 3 (STAT3) activation by suppressing cellular Src kinase (c-Src), Janus kinase 1 (JAK1), Janus kinase 2 (JAK2) and inducing Src homology two phosphatase 2 (SH-PTP2) (Tanigawa et al., 2025). These findings indicate that diosgenin inhibits the STAT3 signaling pathway and may have therapeutic potential in hepatocellular carcinoma (HCC) and other cancers.

Nanotechnology-based approaches have additionally expanded the potential anticancer applications of fenugreek-derived compounds. Nanotechnology has enabled the development of nanoparticle-based systems that can destroy cancer cells while reducing harmful effects on healthy cells (Gmeiner and Ghosh, 2015). Fenugreek extract-mediated zinc oxide nanoparticles (ZnONPs) exhibited cytotoxic effects against different cancer cell lines with reduced toxicity and induced G0/G1 cell cycle arrest when used together with doxorubicin (Radwan et al., 2021).

Additional evidence from colorectal cancer models also supports the anticancer activity of fenugreek-derived products. Two fenugreek protein hydrolysates, namely, Purafect and Esperase, were investigated in colorectal cancer models (Allaoui et al., 2019). The hydrolysates did not affect differentiated Caco2/TC7 cells but reduced proliferation of undifferentiated cells through early apoptosis and G1 cell-cycle arrest, linked to mitochondrial membrane disruption and activates caspase-3. Also, lowered intracellular ROS levels, indicating antioxidant activity (Al-Timimi, 2019).

*In vivo* studies have further supported the anticancer potential of fenugreek and its bioactive constituents in different experimental tumor models. Fenugreek seeds showed protection against rat model of 7,12-dimethylbenz[a]anthracene (DMBA)-induced breast cancer (Amin et al., 2005). Similarly, a related work on pancreatic cancer tested germinated fenugreek seed extract against BXPC-3 cells *in vivo* and *in vitro* (Almalki and Naguib, 2022). In a BXPC-3 mouse model, treatment with the IC_50_ dose of geminated fenugreek seed extract compared with untreated controls, significantly enhanced the survival of pancreatic cancer mice relative to untreated controls and prevented cancer-related damage in pancreatic tissue.

Additional *in vivo* evidence was reported for diosgenin, one of the major bioactive compounds of fenugreek. In an *in vivo* xenograft model, diosgenin administered at 10 mg/kg suppressed tumor growth in nude mice implanted with MCF-7 and MDA-MB-231 breast cancer cells (Liu et al., 2020). Likewise, in a colorectal cancer xenograft model, diosgenin treatment at 100 μ mol/L significantly reduced tumor volume and tumor mass in BALB/c nude mice, which demonstrates antitumor activity against colorectal cancer (Pandey et al., 2025).

Various bioactive compounds and nanomaterials derived from *T. foenum-graecum* exhibited notable anticancer effects in experimental studies. Fenugreek-derived products suppressed proliferation of different cancer cell lines, induced apoptosis and cell-cycle arrest, and regulated cancer-associated pathways including AKT and STAT3, while showing limited toxicity toward normal cells. *In vivo* findings additionally supported the antitumor potential of fenugreek in breast, pancreatic, and colorectal cancer models, highlighting its promise as a natural source for anticancer therapy development.

### 6.7 Hemolytic effect

Hemolytic assays are commonly used to evaluate the blood compatibility and cytotoxicity of biomaterials by measuring hemoglobin release following erythrocyte lysis (Prakash et al., 2025). Materials are categorized as hemolytic when the hemolysis rate exceeds 5%, slightly hemolytic at 2%–5%, and non-hemolytic when the rate is lower than 2% (Tahmasebi and Mohammadi, 2025). Low hemolytic activity is a key indicator of biocompatibility and biosafety in blood-contacting and wound-healing applications.

A polysaccharide isolated from fenugreek exhibits antioxidant properties together with low hemolytic activity (Trabelsi et al., 2025). Fenugreek seed extracts displayed negligible hemolytic toxicity compared with Triton-X-100, with HC50 values reaching 2838 μg/mL (Fatima et al., 2022). Luteolin, a flavonoid identified in fenugreek, binds the pore-forming toxin streptolysin O, inhibiting hemolysis and reducing cytotoxicity in host cells, as illustrated in Figure 3 (Prakash et al., 2025). Fenugreek water-extractable polysaccharides (FWEP) displayed strong antioxidant properties while remaining non-hemolytic toward bovine erythrocytes (Ktari et al., 2017). Fenugreek-derived polysaccharide maintained cell viability above 85% at concentrations up to 100 μg/mL (Trabelsi et al., 2025). FWEP exhibited biological activity by inhibiting radical formation by approximately 73.9% and showed metal-chelating effects up to 88.6% at a concentration of 10 mg/mL (Ktari et al., 2017).

The favorable hemocompatibility of biomaterials derived from fenugreek has facilitated their investigation in hemostatic and wound-healing applications. Galactomannan-based materials have attracted attention because they exhibited good blood compatibility and did not induce erythrocyte damage (Scheibel et al., 2025). The plant-based hydrogel showed only 0.13% hemoglobin release (Tahmasebi and Mohammadi, 2025). Plant-derived polysaccharides have been shown to support wound healing by maintaining good blood compatibility (Trabelsi et al., 2025). Fenugreek extract alone could not form stable hydrogels, but its incorporation into BSA produced mechanically stable composite soft hydrogels and films (Tripathi et al., 2026). Iron and sulfated galactomannan derivatives were reported to be hemocompatible. They did not significantly alter clotting time or induce erythrocyte hemolysis (Scheibel et al., 2025). A fenugreek extract-based nanocomposite hydrogel containing a Pot Marigold extract-loaded copper-based metal-organic framework (PM@HKUST-1) exhibited very low hemolysis, not exceeding 0.6%. This value was lower than those reported for many conventional hemostatic agents. Flavonoid glycosides isolated from the extract were reported to accelerate coagulation by promoting red blood cell and platelet aggregation (Tahmasebi and Mohammadi, 2025).

Fenugreek-derived galactomannans are promising candidates for future blood-contacting, dermo-cosmetic, and biomedical applications (Scheibel et al., 2025; Trabelsi et al., 2025). The non-hemolytic nature and wound healing efficacy of FWEP highlight its promise as a safe biomaterial for skin wound therapy (Ktari et al., 2017). Fenugreek-based hydrogels have been proposed as sustainable, multifunctional materials for skin protection and wound healing, although further clinical validation and scale-up studies are required (Tripathi et al., 2026). Although fenugreek supplementation showed a tendency to reduce gastrointestinal bleeding, larger and longer trials are required (Zarghi et al., 2021). Table 2 summarizes the pharmacological effects of fenugreek.

**TABLE 2:** Pharmacological properties of fenugreek.

Pharmacological activities	Plant part	Effects	Model	References
Antioxidant	Seed	Strong free-radical scavenging activity (RSA)	*In vitro*: DPPH and ABTS radical scavenging	Akbari et al. (2019)
		Enhanced hepatic antioxidant defense by increasing SOD activity	*In vivo*: 12 month-old aging mice	Tewari et al. (2020)
		Increased antioxidant enzymes and decreased MDA in heart tissue	*In vivo*: Alcohol-intoxicated Wistar rats	Sekhar et al. (2024)
		DNA damage protection against Fenton-induced oxidative stress	*In vitro*: DNA protection assay	Dhull et al. (2020)
		Restored antioxidant defense by enhancing hepatic enzymatic antioxidants	*In vivo*: Rats exposed to bisphenol A	Guardiola et al. (2018)
Anticancer	Seed	Prevents metastasis in pancreatic cancer cells	*In vitro*: Panc-1, Miapaca-2, SNU-213, Aspc-1, 293T	Morshidi et al. (2025)
		Improves survival rate and protects pancreatic tissue	*In vitro*: BXPC-3 cells *In vivo*: Swiss albino mice	Almalki and Naguib (2022)
		Inhibiting the role of cathepsin G in promoting breast cancer cell progression and reducing PAFAH1B2	*In vitro*: MCF-7 cells, HL-60 lysates	Tanigawa et al. (2025)
		Inducing apoptosis, suppressing tumor growth, lowering ascitic volume	*In vitro*: EAC, MCF-7, HCT-116, HEPG2, WISH *In vivo*: Swiss albino mice with Ehlich ascites carcinoma	Radwan et al. (2021)
		Selective inhibition of colorectal cancer cell proliferation *via* early apoptosis induction, reduction of intracellular ROS	*In vitro*: Caco2/TC7 human colorectal adenocarcinoma cells	Allaoui et al. (2019)
Antidiabetic	Seed	Strong α -glucosidase (95.24%) inhibition and insulinotropic activity	*In vitro*: Enzyme assays *In vivo*: Wistar rats (OGTT, OSTT, IPGTT, STZ-diabetic model)	Laila et al. (2023)
		Reduces TC, TG, and LDL-C, strong improvement of lipid metabolism	Human clinical study	Geberemeskel et al. (2019)
		Increases serum insulin, lowers TC, urea, uric acid, TG	*In vivo*: STZ-induced diabetic Wistar rats	Eidi et al. (2007)
		Improved glucose metabolism by a reduction in fasting plasma glucose	*In vivo*: STZ-induced diabetic rats	Jiang et al. (2018)
		Lowers blood glucose and HbA1c, improves insulin sensitivity and hepatic, muscle glycogen	*In vivo*: STZ-induced type 2 diabetic Wistar rats	Subramanian and Prasath (2014)
Antilipidemic	Seed	Trigonelline normalized the lipid profile, amelioration of serum cholesterol, TG and FFAs	*In vivo*: HFD/STZ-induced type 2 diabetic Wistar rats	Subramanian and Prasath (2014)
		Saponin effectively accelerate cholesterol metabolism, inhibited cholesterol synthesis and RCT process	*In vivo*: HFD-induced dyslipidemic Sprague-Dawley rats	Chen et al. (2017)
​	​	Polyphenol stilbenes reduced lipid accumulation and inhibited cellular triglyceride accumulation in adipocytes	*In vitro*: Insulin-resistant 3T3-L1 adipocytes	Li et al. (2018)
		Improved HDL to LDL ratios	*In vivo*: C57BL/6J mice fed a HFD	Knott et al. (2017)
	Leaf extract/powder	Upregulation of LDLR gene expression helped improve dyslipidemia	*In vivo*: HCD-fed male hamsters	Kassaee et al. (2021)
Anti-inflammatory	Seed	↓ TNF- α , IL-6, PGE2 and NO levels, infiltration of inflammatory cells	*In vitro*: RAW 264.7 macrophages *In vivo*: Carrageenan-induced rat models	Fatima et al. (2022)
		↓ NO, TNF- α and IL-6 production ↑ IL-10 levels	*In vitro*: LPS-activated THP-1-derived macrophages	Kmail et al. (2022)
		↓ carregeenan-induced paw oedema; up to 63.23% inhibition of inflammation at 200 mg/kg	*In vivo*: Swiss albino mice paw-oedema model	Cheurfa et al. (2022)
		↓ LOX (IC_50_ = 19.69 μ g/mL) and HYA (IC_50_ = 17.57 μ g/mL)	*In vitro*: Enzyme inhibition assays	Neagu et al. (2023)
		↓ IL-6, IL-1 β, and TNF- α ↑ IL-10 and IL-4 Reduced adipose inflammation	*In vitro*: LPS-activated macrophages *In vivo*: HFD-induced C57BL/6 mice	Sen et al. (2025)
Antibacterial and antifungal	Seed	4-HIL exhibited the maximum inhibition of fungal growth 69.9% against *M. oryzae*	*In vitro*: antifungal assay and greenhouse *in vivo* rice plants	Gajbar et al. (2022)
		Chitosan-mediated 2.5% nanocomposite showed fungal growth at 521 CFU/mL and significantly reduced *C. albicans* colony formation	*In vitro*: denture tissue conditioner model in artificial saliva	Damle et al. (2025)
		The AgNPs exerted significant antimicrobial activity	*In vitro*: MIC assays on bacteria and fungi	Varghese et al. (2019)
	Leaf extract	The TF-TiO_2_NPs exhibited antimicrobial activity with inhibition zones of about 8.5–11.5 mm	*In vitro*: disc-diffusion assay	Subhapriya and Gomathipriya (2018)
		The Ag-ZnONPs showed strong antimicrobial and antioxidant activities	*In vitro*: disk-diffusion, MIC/MBC/MFC assays	Noohpisheh et al. (2020)
Hemolytic	Seed	No hemolysis was observed toward bovine erythrocytes at 1–1000 μ g/mL	*In vitro*: hemolysis assay on erythrocytes	Ktari et al. (2017)
		Hemolysis ratio did not exceed 0.6%; hydrogel alone showed 0.13% hemoglobin release	*In vitro*: human RBC hemolysis assay	Tahmasebi and Mohammadi (2025)

### 6.8 Safety profile and toxicological considerations

Although *T. foenum-graecum* has a long history of dietary and medicinal use, comprehensive evaluation of its safety profile remains essential to support its increasing application in nutraceuticals, functional foods, and phytopharmaceutical products. Several preclinical and clinical studies have investigated the toxicological properties of fenugreek and generally reported favorable safety outcomes. A systematic review employing the Toxicological Data Reliability Assessment Tool (ToxRTool) evaluated 45 preclinical and clinical toxicity studies of fenugreek seeds. Among the studies assessed, 17 were classified as “reliable without restrictions”, indicating a broad margin of safety for standardized fenugreek seed preparations (Kandhare et al., 2019). Clinical evidence also supports the safety of fenugreek-derived preparations. In a randomized double-blind placebo-controlled study involving 60 healthy male subjects receiving furostanol glycosides-based fenugreek seed extract (Fenu-FG; 600 mg/day) for 8 weeks, no serious adverse events were reported, and biochemical and hematological parameters remained within normal physiological ranges, indicating that the preparation was safe and well tolerated (Wankhede et al., 2016). In a 7-week rat study, dietary supplementation with fenugreek seeds (10% of the diet) did not induce significant alterations in biochemical, hematological, hepatic, or renal parameters (Mbarki et al., 2017).

Several standardized fenugreek seed preparations have undergone acute and sub-chronic toxicity evaluation in accordance with Organisation for Economic Co-operation and Development (OECD) guidelines. Glycoside-based standardized fenugreek seed extract (SFSE-G) was found safe for acute and sub-chronic administration in rats with no mutagenic potential. Similarly, IDM01, a standardized fenugreek formulation enriched with 4-hydroxyisoleucine and trigonelline, was reported to be safe without mutagenicity or genotoxicity (Kandhare et al., 2019). A more detailed toxicological evaluation of IDM01 further confirmed its safety. Acute oral administration at 2000 mg/kg produced no mortality, treatment-related adverse effects, or pathological abnormalities, indicating an LD50 greater than 2000 mg/kg. In addition, repeated administration for 90 days at doses up to 1000 mg/kg/day did not result in treatment-related toxicity, and the no-observed-adverse-effect level (NOAEL) was established at 500 mg/kg/day (Deshpande P. O. et al., 2017). In addition, low molecular weight galactomannan-based fenugreek seed extract (LMWGAL-TF) was found safe during acute and sub-chronic toxicity studies with no evidence of mutagenicity (Kandhare et al., 2019).

The mutagenic and genotoxic potential of standardized fenugreek seed preparations has been evaluated using several validated assays. The mutagenic potential of glycoside-based standardized fenugreek seed extract (SFSE-G) was evaluated using the Ames bacterial reverse mutation assay, which demonstrated no evidence of mutagenicity in any tested *Salmonella typhimurium* strain, either with or without metabolic activation (Deshpande P. et al., 2017). Similarly, IDM01 showed no evidence of mutagenicity up to 5000 μg/plate in the Ames assay and did not induce structural chromosomal aberrations up to 50 mg/culture, further supporting the lack of mutagenic and genotoxic concerns associated with standardized fenugreek preparations (Deshpande P. O. et al., 2017). Further support for the genoprotective potential of fenugreek was provided by FWEP, which was shown to protect DNA against hydroxyl radical-induced damage and reduce thiamethoxam-induced DNA degradation and genotoxicity *in vitro* and *in vivo* (Feki et al., 2019).

Despite the generally favorable safety profile reported for standardized fenugreek preparations, some studies have raised concerns regarding reproductive toxicity. In pregnant mice, administration of lyophilized aqueous extract from fenugreek seeds at doses of 500 and 1000 mg/kg/day throughout gestation reduced litter size from 11.2 pups in the control group to 8.75 and 4.8 pups, respectively. In addition, offspring body weight at birth decreased from 4.01 g in controls to 2.74 and 2.93 g, respectively, and several morphological abnormalities were observed, suggesting potential fetotoxic and teratogenic effects at high doses (Khalki et al., 2010). Therefore, caution may be warranted regarding the use of fenugreek-derived products during pregnancy until further safety data become available.

Reported adverse effects of fenugreek in clinical studies are generally mild and include diarrhea, flatulence, dyspepsia, abdominal discomfort, dizziness, and hypoglycemia, particularly at high doses or during prolonged administration (Ouzir et al., 2016). Fenugreek may interact with certain medications. For example, concurrent use with insulin or oral antidiabetic drugs may increase the risk of hypoglycemia, while co-administration with anticoagulant or antiplatelet agents may increase the risk of bleeding (Arunabha and Bhattacharjee, 2019). Rare allergic reactions have also been reported following fenugreek consumption, and limited evidence suggests that fenugreek may affect potassium and thyroid hormone levels (Yadav and Baquer, 2014). Consistent with these observations, fenugreek has been recognized as a potential food allergen, with reported cases of rhinitis, asthma, bronchospasm, urticaria, angioedema, and anaphylaxis. Cross-reactivity has also been observed in individuals allergic to peanuts and chickpeas (Ouzir et al., 2016).

## 7 Conclusion and future directions

Fenugreek is a plant of considerable nutritional, phytochemical, and pharmacological importance, with diverse applications in human health, food systems, and sustainable agriculture. The available evidence indicates that its diverse biological activities are largely attributed to bioactive compounds such as saponins, flavonoids, alkaloids, galactomannans, and amino acid derivatives. Among the reported pharmacological effects, the strongest evidence supports its antidiabetic, antioxidant, anti-inflammatory, and antilipidemic activities, which have been demonstrated in numerous experimental studies and supported by a limited number of clinical investigations.

Despite these promising findings, important limitations remain. Most evidence is derived from *in vitro* and animal studies, while clinical studies are relatively scarce and often limited by small sample sizes, short intervention periods, and variability in formulations and dosages. Furthermore, differences in phytochemical composition among fenugreek preparations highlight the need for standardization. Although current data generally indicate a favorable safety profile, additional studies are required to establish long-term safety and clarify potential toxicological concerns and herb − drug interactions.

Future research should increasingly focus on turning its well-documented nutritional and pharmacological potential into standardized, evidence-based applications in functional foods and nutraceuticals. Although fenugreek has a long history of safe use, the variability in its bioactive constituents due to genotype, growing conditions, processing, and formulations remains a major challenge. Further breeding programs associated with molecular markers, omics tools, and genome-editing technologies are essential to develop high-yielding, metabolite-rich, and stress-resilient cultivars, which can be used for specific health and industrial applications.

Despite extensive preclinical evidence, clinical and mechanistic data remain insufficient to fully support many of the reported biological activities. Many of these findings still lack confirmation from well-designed human studies and therefore require further investigation. Rigorous, long-term randomized controlled trials are required to justify these effects and elucidate dose-response connections. A parallel, thorough examination of specific bioactive compounds such as trigonelline, diosgenin, 4-HIL, and less-explored constituents is crucial to clarify structure − activity correlations, synergistic or antagonistic interactions, and molecular targets. Furthermore, the integration of metabolomics, transcriptomics, and gut microbiota analyses.

Ultimately, technological advancements will notably impact the future of fenugreek-based products. Advanced extraction, nano-delivery systems, and utilization of compounds derived from fenugreek present intriguing strategies. However, these improvements must be complemented by large-scale safety assessments, standardized analytical methods, and well-defined regulatory frameworks. In addition, further toxicological investigations and harmonized quality-control standards will be essential to facilitate the clinical translation and regulatory approval of fenugreek-derived products. Maximizing fenugreek’s value in modern nutrition and healthcare depends on a multidisciplinary strategy that unites plant breeding, food science, pharmacology, and clinical research.
